# *Bt* Rice Expressing Cry2Aa Does Not Harm *Cyrtorhinus lividipennis*, a Main Predator of the Nontarget Herbivore *Nilapavarta lugens*

**DOI:** 10.1371/journal.pone.0112315

**Published:** 2014-11-06

**Authors:** Yu Han, Jiarong Meng, Jie Chen, Wanlun Cai, Yu Wang, Jing Zhao, Yueping He, Yanni Feng, Hongxia Hua

**Affiliations:** 1 Hubei Insect Resources Utilization and Sustainable Pest Management Key Laboratory, College of Plant Science and Technology, Huazhong Agricultural University, Wuhan, P.R. China; 2 College of Life Science and Technology, Huazhong Agricultural University, P.R. China; Ghent University, Belgium

## Abstract

T2A-1 is a newly developed transgenic rice that expresses a synthesized *cry2Aa* gene driven by the maize *ubiquitin* promoter. T2A-1 exhibits high resistance against lepidopteran pests of rice. The brown planthopper, *Nilapavarta lugens* (Stål), is a main nontarget sap-sucking insect pest of rice, and *Cyrtorhinus lividipennis* (Reuter) is the major predator of the eggs and young nymphs of planthoppers. As *C. lividipennis* may expose to the Cry2Aa protein via *N. lugens*, it is therefore essential to assess the potential effects of transgenic *cry2Aa* rice on this predator. In the present study, three experiments were conducted to evaluate the ecological risk of transgenic *cry2Aa* rice to *C. lividipennis*: (1) a direct feeding experiment in which *C. lividipennis* was fed an artificial diet containing Cry2Aa at the dose of 10-time higher than that it may encounter in the realistic field condition; (2) a tritrophic experiment in which the Cry2Aa protein was delivered to *C. lividipennis* indirectly through prey eggs or nymphs; (3) a realistic field experiment in which the population dynamics of *C. lividipennis* were investigated using vacuum-suction. Both direct exposure to elevated doses of the Cry2Aa protein and prey-mediated exposure to realistic doses of the protein did not result in significant detrimental effects on the development, survival, female ratio and body weight of *C. lividipennis*. No significant differences in population density and population dynamics were observed between *C. lividipennis* in transgenic *cry2Aa* and nontransgenic rice fields. It may be concluded that transgenic *cry2Aa* rice had no detrimental effects on *C. lividipennis*. This study represents the first report of an assessment continuum for the effects of transgenic *cry2Aa* rice on *C. lividipennis*.

## Introduction

Rice, *Oryza sativa* L., is the staple food of more than three billion people of Asia [1]. More than 200 species of insect pests infest rice during its growing season [2], and this causes 15% to 25% yield losses of rice [3], [4]. Among these insects, lepidopteran species such as stem borers and leaffolders are particularly serious chronic pests and cause large annual yield losses [5], [6]. Traditional management of lepidopteran pests is mainly dependent on the spraying of pesticides. However, excessive or continual applications of pesticide not only cause environmental contamination and the resurgence of herbivores but also reduce populations of the natural enemies of these herbivores [7], [8]. Researchers have therefore been encouraged to seek more effective and environmentally friendly methods to control lepidopteran pests.

The *Bacillus thuringiensis* (*Bt*) insecticidal δ-endotoxin has been used as a biological insecticide for more than 50 years, and it is now possible to introduce different *Bt* genes into rice. Transgenic rice plants expressing Bt proteins have been shown to be effective against many lepidopteran insect pests [9] and have led to great reductions in the use of insecticides [10], [11]. A series of rice lines that express various *Bt* genes (e.g., *cry1Ab*, *cry1Ab/1Ac*, *cry1C* and *cry2A*) have been developed and can effectively suppress the infestation of target lepidopteran insect pests [12]–[14]. Despite these successes, concerns have been raised regarding the potential impacts of transgenic *Bt* rice on nontarget herbivores and their natural enemies through tritrophic transmission. It is therefore necessary to conduct an environmental risk assessment of any novel transgenic *Bt* rice prior to its commercialization, and the environmental risks of each rice line must be evaluated on a case-by-case basis.

The brown planthopper, *Nilapavarta lugens* (Stål) (Hemiptera: Delphacidae), is the main nontarget sap-sucking insect pest of rice and causes significant annual reductions in the rice yield [15], [16]. The evaluation of nontarget effects of transgenic rice on the natural enemies of *N. lugens* is an important part of the environmental risk assessment, and should be conducted before the commercialization of any novel *Bt* rice. Several previous reports have examined the effects of transgenic *Bt* rice on the predators of *N. lugens*. Although the Cry1Ab protein can be transferred from transgenic rice plants to predators via *N. lugens*, no adverse effects have been found on any of the fitness parameters (survival, developmental time, weight and fecundity) of numerous predators [*Cyrtorhinus lividipennis* (Reuter) (Hemiptera: Miridae), *Pardosa pseudoannulata* (Bösenberg et Strand) (Araneae: Lycosidae), *Pirata subpiraticus* (Bösenberg et Strand) (Araneae: Lycosidae), *Propylea japonica* (Thunberg) (Coleoptera: Coccinellidae) and *Ummeliata insecticeps* (Bösenberg et Strand) (Araneae: Linyphiidae)] that preyed on *N. lugens* reared on *Bt*-transformed rice lines in the laboratory [17]–[22]. Additionally, the results from field experiments have also shown that the population densities and population dynamics of *U. insecticeps*, *C. lividipennis* and *P. pseudoannulata* are not significantly different between transgenic *cry1Ab* rice and non-*Bt* rice fields [21]–[23]. All of these results have indicated that the predators of *N. lugens* are not affected by the Cry1Ab protein. T2A-1 is a relatively newly developed transgenic rice line expressing *cry2Aa* driven by the maize *ubiquitin* promoter, and it exhibits high resistance against lepidopteran pests of rice [12]. However, the potential effects of this rice line on the predators of *N. lugens* have received little attention. T2A-1 did not cause direct detrimental effects on the larvae of *Chrysoperla sinica* (Tjeder) (Neuroptera: Chrysopidae), a general predator of *N. lugens* [24]; that study represents the only existing report about the effects of Cry2Aa on the predators of *N. lugens*.

*Cyrtorhinus lividipennis* (Reuter) (Hemiptera: Miridae) is a major predator of the eggs and young nymphs of planthoppers, and it is a primary factor regulating the population density of *N. lugens* in rice fields [7], [25], [26]. Thus, *C. lividipennis* may expose to the Bt protein via *N. lugens*. The potential effects of transgenic *Bt* rice on *C. lividipennis* should be evaluated. Transgenic *cry1Ab* rice had no negative effects on the life-table parameters, population density and population dynamics of *C. lividipennis* [18], [23]. However, no such study of transgenic *cry2Aa* rice has yet been conducted with *C. lividipennis*. In the current study, we conducted comprehensive experiments in the laboratory and in rice fields to examine the effects of T2A-1 on *C. lividipennis*. The effects of T2A-1 via prey on the life-table parameters and the functional response of *C. lividipennis* to *N. lugens*, and the direct toxicity of the Cry2Aa protein to *C. lividipennis* were evaluated in the laboratory. The effects of T2A-1 on population density and population dynamics were investigated through a 3-year experiment in rice fields. ELISA was used to determine whether the Cry2Aa protein could be transferred to *C. lividipennis* via *N. lugens*.

## Materials and Methods

### Ethics Statement

All necessary permits were obtained for the described field studies. Permission of small-field test of the transgenic line (T2A-1) at the suburbs of Xiaogan City and the suburbs of Suizhou City during the 2011-2013 was issued by Ministry of Agriculture of the People's Republic of China. Contact: Hao Chen; Phone: +86 27 87280516.

### Plant materials

The transgenic *Bt* rice line (T2A-1) and the nontransgenic parental *indica* rice line Minghui 63 were selected for the experiments. T2A-1 expresses a synthesized *cry2Aa* gene driven by the maize *ubiquitin* promoter. T2A-1 is homozygous and exhibits high resistance against lepidopteran pests of rice [12]. Minghui 63 is an elite *indica* restorer strain, and served as nontransgenic control. Both rice lines were gifted by National Key Laboratory of Crop Genetic Improvement, Wuhan, China.

The two rice lines used for the laboratory experiments were cultured in different plastic tanks (25 cm length×20 cm width×3 cm height) in Yoshida culture solution [27]. Fifteen-day-old rice seedlings (approximately 15 cm in height) were used in the experiments. All plants were maintained at 26°C±2°C, and the relative humidity was approximately 80%.

### Insects for the laboratory experiments

The original adults of *N. lugens* were collected from paddy fields in Wuhan, Hubei Province, China. Prior to the tritrophic bioassay, independent colonies of *N. lugens* were established on T2A-1 and Minghui 63 and maintained for more than ten generations. The *C. lividipennis* individuals were collected from paddy fields in Xiaogan and reared with eggs of *N. lugens* infested on Minghui 63. The colony was maintained for more than six generations before its use in the present study. The insects were cultured at 28±1°C, RH 70±5% and with a light-dark cycle of 14 h:10 h. Both susceptible strains of *Plodia interpunctella* (Hubner) (Lepidoptera: Pyralidae) and *Cnaphalocrocis medinalis* (Guenee) (Lepidoptera: Pyralidae) were used to confirm the bioactivity of the Cry protein.

### Prey egg–mediated effects of transgenic *cry2Aa* rice on the life-table parameters of *C. lividipennis*

Two reproductive females of *N. lugens* were placed into a glass tube (3 cm diameter×25 cm length) containing six or seven 15-day-old rice seedlings (Minghui 63 and T2A-1) and allowed to lay eggs for 2 days. The *N. lugens* adults were then removed, and the eggs of *N. lugens* on the rice seedlings were used as the prey of *C. lividipennis*. Newly hatched nymphs of *C. lividipennis* (<24 h) were placed individually in glass tubes. All tubes were sealed with nylon mesh. During the period from the first to the third instar of *C. lividipennis*, the rice seedlings were refreshed every 2 days, and during the period from the fourth instar to adulthood, the rice seedlings were refreshed every day. Yoshida culture solution [27] was used to keep the rice seedlings fresh. The survival and molting of the *C. lividipennis* nymphs were recorded every day. After the *C. lividipennis* adults emerged, the sex and body weight of these adults were recorded. Thirty-two nymphs of *C. lividipennis* were tested for each rice line.

### Prey nymph–mediated effects of transgenic *cry2Aa* rice on the life-table parameters of *C. lividipennis*

Newly molted second-instar nymphs (<24 h) of *C. lividipennis* were placed individually in glass tubes (2 cm diameter×12 cm length) covered with tampons. The bottom of each tube was filled with a piece of wetted sponge to maintain humidity. Newly hatched nymphs of *N. lugens* (24–48 h after hatching), reared on either Minghui 63 or T2A-1 rice plants, were employed as the prey of *C. lividipennis*. During the period from the second to the third instar of *C. lividipennis*, 10 nymphs of *N. lugens* were provided daily, and during the period from the fourth instar to adulthood, 20 nymphs were provided daily. The survival and molting of the *C. lividipennis* nymphs were monitored on a daily basis. After the adults of *C. lividipennis* emerged, the sex and body weight of these adults were recorded. Eighty nymphs of *C. lividipennis* were tested for each rice line.

### Cry2Aa contents in rice plants, *N. lugens* and *C. lividipennis*

The sheaths of 15-day-old rice seedlings, neonates of *N. lugens* fed on T2A-1 or Minghui 63 for 2 days, *N. lugens* eggs laid by *N. lugens* adults fed on T2A-1 or Minghui 63, and third- or fourth-instar nymphs of *C. lividipennis* that preyed on eggs or nymphs of *N. lugens* fed on T2A-1 or Minghui 63 were collected. Four or five samples were collected for each treatment. Before the assay, the insect samples were washed four times with PBST (PBS/0.55% Tween-20) to remove any Cry protein from their outer surfaces. The Cry protein contents were determined using AP005 ENVIRONLOGIX kits (ENVIRONLOGIX, USA). The kits were used according to the manufacturer's instructions.

### Exposure of *C. lividipennis* to Cry2Aa at high dose

Lyophilized Cry2Aa protein was purchased from the Biochemistry Department Laboratory, School of Medicine, Case Western Reserve University, USA. The Cry2Aa protoxin was expressed by *Escherichia coli*, then the protoxin inclusion bodies were solubilized and trypsinized, subsequently the toxins were purified and lyophilized. The purity of toxins is about 95–98%, and the molecular size of activated toxin is 65 kDa. Potassium arsenate (PA, KH_2_AsO_4_), which has been previously reported as toxic to insects [24], [28], was used as a toxic model compound and was purchased from Sigma-Aldrich (St. Louis, MO).

The results of the preliminary experiments showed that the chemically defined diet for *N. lugens* as described by Fu et al. [29] was sufficient for sustaining the growth and development of *C. lividipennis* from the second instar to adulthood (The survival of *C. lividipennis* from the first instar to adulthood was low). The artificial diet for *N. lugens* was therefore used as the medium to deliver the Cry2Aa protein to the gut of *C. lividipennis*. Second-instar nymphs of *C. lividipennis* were reared on one of three different diets: i) an artificial diet (negative control); ii) an artificial diet containing 300 µg/ml of Cry2Aa (a level over ten times higher than that to which *C. lividipennis* would realistically be exposed in rice fields); iii) an artificial diet containing 40 µg/ml of PA (positive control). The diets were refreshed daily. The molting and survival of *C. lividipennis* were observed daily. When the adults emerged, their genders and body weights were recorded. Thirty-six nymphs of *C. lividipennis* were evaluated for each treatment.

To ensure the stability of the Cry2Aa protein in the artificial diets before and after 24 h of feeding exposure, the Cry2Aa proteins were extracted from the artificial diets, and the concentrations of Cry2Aa protein in the diet were determined using AP005 ENVIRONLOGIX kits.

To examine the bioactivity of this batch of Cry2Aa on lepidopteran insects, Cry2Aa was mixed with an artificial diet for *P. interpunctella*. Each bioassay included five concentrations for Cry2Aa (2, 10, 20, 30, 40 µg/g) plus a control. Forty newly hatched *P. interpunctella* larvae javascript:void(0); were introduced into the artificial diets of each concentration and five replicates were tested. Mortality of the larvae was determined after 1 week. The LC_50_ (concentration resulting in 50% *P. interpunctella* larval mortality) of this batch of Cry2A protein was measured.

To examine the bioactivity of Cry2Aa protein in the artificial diets before and after 24 h of feeding exposure, the artificial diets containing Cry2Aa were appropriately diluted and sprinkled on the leaves of corn. After 2 h of air-drying, 15 *Bt*-susceptible second-instar larvae of *C. medinalis* were distributed onto the leaves for each treatment. Four replicates were tested in each treatment. Mortality of the insects were recorded 48 h later.

### Effects of transgenic *cry2Aa* rice on the functional response of female *C. lividipennis*

*C. lividipennis* individuals were reared on T2A-1 or Minghui 63 rice plants infested with *N. lugens* eggs for one generation. Female adults of *C. lividipennis* (2 days after eclosion) were starved for 24 h, and each female was then transferred to one glass tube (2 cm diameter×12 cm length) containing three or four 15-day-old rice seedlings infested with first-instar nymphs of *N. lugens*. The experimental densities of *N. lugens* were 10, 20, 30, 40 and 50 nymphs per tube. The number of *N. lugens* consumed by *C. lividipennis* was recorded after 24 h. The experiment was repeated five times for each density.

### Field experiment design

The experiments were conducted during the growing seasons of 2011–2013 at the two different sites, where field trials of *Bt* rice were permitted, in Hubei Province, China. The first site located in the suburbs of Xiaogan City, and the second site located in the suburbs of Suizhou City. The layout of the plots in the field followed a completely randomized block design with four replications for each rice line. Each experimental plot was 150 m^2^ (10 m×15 m) and was surrounded by a 1 m wide unplanted border. The entire experimental field was bordered by five rows of the nontransgenic control plants. The *Bt* and non-*Bt* control rice lines were sown in early May and transplanted 1 month after sowing. The seedlings were manually transplanted with one seedling per pot, 13.3 cm between plants within a row, and 29.9 cm between rows. The agronomic practices used for growing the rice, including fertilization and irrigation, were consistent with those followed by local farmers, except that no insecticides were applied during the whole growing season.

### Field sampling with a vacuum-suction machine

Sampling of *C. lividipennis* was conducted as described by Xu et al. [30]. Arthropods at both field sites were collected using a vacuum-suction machine, constructed basing on a description by Carino et al. [31] and supplemented by a square sampling box (50 cm length×50 cm width×120 cm height) with a metal frame enclosed by Mylar film. Samples were collected every 10–15 days, starting 1 month after transplantation and continuing until the rice was ripe (as measured by grain maturity and harvest). On each sampling date, a square sampling box was placed at random along the diagonal line of each test plot at each site, with five subsamples per plot. The sample locations in each plot were marked with bamboo stakes to avoid resampling at the same location. Arthropods inside the frame enclosure were collected using the vacuum-suction machine for 5 min at each sampling location and were preserved in 75% ethanol. All samples were taken back to the laboratory and identified to the species level.

### Data analysis

ELISA data and body weights were compared using the Student's *t*-tests. The Chi-square test was used for the parameters of preimaginal survival and female ratio. Nymphal developmental time was analyzed using Mann–Whitney *U*-tests, as the data did not fulfill the assumptions required for parametric analyses (normal distribution of residues and homogeneity of error variances). Survival response to the artificial diets containing Cry2Aa was analyzed using the Kaplan-Meier procedure, and the log-rank test was used in the purified toxin experiment.

In the field experiment, the population density and population dynamics determined by vacuum-suction were used to evaluate the impacts of the transgenic *Bt* rice on *C. lividipennis* populations in the fields. The population density of *C. lividipennis* was represented by seasonal means as captured by vacuum-suction. The population dynamics of the predators were measured by the means at each sampling date. Population density and population dynamics were analyzed using the Student's *t*-test.

The data from the functional response experiment were fitted to Holling's “Type II” disc equation, which estimates *Na* as follows: *Na*  =  *aTN/*(1 + *aThN*), where *Na* is the number of prey attacked, *N* is prey density, and *T* is the duration of the experiment (*T* = 1 day in the present study). The parameters *a* (instantaneous search rate) and *Th* (time required to handle a prey item) were calculated via least-squares nonlinear regression based on the Gauss-Newton method.

The percentage data were arcsine–square root transformed, and all count data were square root (*x*+1) or log _10_ (*x*+1) transformed before being subjected to data analysis. The untransformed means are presented in the results. All statistical analyses were performed using the software package SPSS (version 16.0 for Windows, 2007).

## Results

### Prey-mediated effects of transgenic *cry2Aa* rice on the life-table parameters of *C. lividipennis*

No significant differences were observed between the developmental time, preimaginal survival, female ratio and fresh body weight of *C. lividipennis* adults reared with eggs or nymphs of *N. lugens* fed on *Bt* and non-*Bt* rice (*P*>0.05) (Tables 1, 2).

**Table 1:** Prey-mediated effects of Cry2Aa on life-table parameters of *Cyrtorhinus lividipennis* preying eggs of *Nilapavarta lugens* reared with T2A-1 or Minghui 63 rice plants.

Parameters	T2A-1	Minghui 63	Statistics
1st instar developmental time (days ± SE)^a^	2.3±0.09 (32)	2.5±0.11 (32)	*U* = 415, *P* = 0.117
2nd instar developmental time (days ± SE)^a^	1.7±0.09 (30)	1.6±0.10 (30)	*U* = 400.5, *P* = 0.387
3rd instar developmental time (days ± SE)^a^	1.5±0.09 (29)	1.5±0.10 (30)	*U* = 435, *P* = 1.000
4th instar developmental time (days ± SE)^a^	1.8±0.09 (29)	1.8±0.07 (30)	*U* = 400, *P* = 0.473
5th instar developmental time (days ± SE)^a^	2.7±0.09 (29)	2.9±0.14 (29)	*U* = 364, *P* = 0.399
Whole nymphal stage developmental time (days ± SE)^a^	10.0±0.13 (28)	10.3±0.13 (29)	*U* = 313, *P* = 0.080
Preimaginal survival (%)^b^	87.5	90.6	*χ* ^2^ = 0.002, *P* = 0.967
Female ratio (%)^b^	42.9	55.2	*χ* ^2^ = 0.864, *P* = 0.352
Male weight (mg ± SE)^c^	0.55±0.03	0.49±0.03	*t* = −1.492, *P* = 0.179
Female weight (mg ± SE)^c^	0.93±0.02	0.92±0.04	*t* = −0.193, *P* = 0.851

The experiment started with 32 nymphs per treatment. (n), number of individuals at each development stage.

a Mann–Whitney *U*-test.

b Chi-square test.

c Student's *t*-test.

**Table 2:** Prey-mediated effects of Cry2Aa on life-table parameters of *Cyrtorhinus lividipennis* preying nymphs of *Nilapavarta lugens* reared with T2A-1 or Minghui 63 rice plants.

Parameters	T2A-1	Minghui 63	Statistics
2nd instar developmental time (days ± SE)^a^	1.9±0.08 (76)	1.8±0.06 (77)	*U* = 2.538E3*, *P* = 0.102
3rd instar developmental time (days ± SE)^a^	1.8±0.07 (63)	2.0±0.08 (70)	*U* = 1.875E3*, *P* = 0.087
4th instar developmental time (days ± SE)^a^	2.4±0.09 (50)	2.4±0.13 (48)	*U* = 1.180E3*, *P* = 0.873
5th instar developmental time (days ± SE)^a^	2.8±0.17 (29)	2.7±0.15 (27)	*U* = 367.5, *P* = 0.654
2nd instar-adult developmental time (days ± SE)^a^	8.8±0.21 (28)	9.2±0.20 (26)	*U* = 292, *P* = 0.190
Preimaginal survival (%)^b^	35.0	32.5	*χ* ^2^ = 0.112, *P* = 0.738
Female ratio (%)^b^	42.9	38.5	*χ* ^2^ = 0.108, *P* = 0.743
Male weight (mg ± SE)^c^	0.33±0.05	0.30±0.01	*t* = −0.420, *P* = 0.696
Female weight (mg ± SE)^c^	0.46±0.02	0.43±0.09	*t* = −0.322, *P* = 0.764

The experiment started with 80 nymphs per treatment. (n), number of individuals at each development stage.

*E3 = 10^−3^.

a Mann–Whitney *U*-test.

b Chi-square test.

c Student's *t*-test.

The Cry2Aa content in T2A-1 rice sheaths was 13.9±1.2 µg/g. The content of Cry2Aa in nymphs of *N. lugens* fed on T2A-1 was 5.3±0.8 ng/g, only 0.04% of that in the rice sheaths. The content of Cry2Aa in the eggs of *N. lugens* fed on T2A-1 was undetectable (Table 3).

**Table 3:** Contents of Cry2Aa protein in rice sheath tissue, *Nilapavarta lugens* and *Cyrtorhinus lividipennis*.

Treatments	T2A-1	Minghui 63
Sheath of rice plants	13.9±1.2 µg/g	Not detectable
Eggs of *N.lugens*	Not detectable	Not detectable
Nymphs of *N. lugens*	5.3±0.8 ng/g	Not detectable
*C. lividipennis* provided with *N. lugens* eggs with rice plants	200.2±33.2 ng/g	Not detectable
*C. lividipennis* provided with *N. lugens* nymphs with rice plants	48.5±13.0 ng/g	Not detectable
*C. lividipennis* provided with *N. lugens* eggs without rice plants	Not detectable	Not detectable
*C. lividipennis* provided with *N. lugens* nymphs without rice plants	Not detectable	Not detectable
*C. lividipennis* provided with rice plants	17.1±8.6 ng/g	Not detectable

Data are represented as mean ± SE.

When *N. lugens* nymphs and T2A-1 seedlings were provided simultaneously to *C. lividipennis*, Cry2Aa was detectable, and the concentration of Cry2Aa in *C. lividipennis* was 48.5±13.0 ng/g; this value was much lower than that in the rice sheaths (13.9±1.2 µg/g) but higher than that in the nymphs of *N. lugens* (5.3±0.8 ng/g). However, when the *N. lugens* nymphs fed on T2A-1 were removed from T2A-1 and the nymphs alone were provided to *C. lividipennis*, no Cry2Aa was detected in the predator. When *N. lugens* eggs and T2A-1 seedlings were provided to *C. lividipennis* simultaneously, Cry2Aa was detectable, and the concentration of Cry2Aa in *C. lividipennis* was 200.2±33.2 ng/g. However, when the *N. lugens* eggs were removed from the T2A-1 seedlings and the eggs alone were provided to *C. lividipennis*, Cry2Aa was not detectable in the predator. As was expected, no Cry2Aa protein was detected in the Minghui 63 rice plants (Table 3).

### Purified Cry2Aa protein bioassay

Before and after exposure to *C. lividipennis* for 24 h, the concentrations of Cry2Aa in the artificial diets were 98.4±3.2 µg/g and 91.1±3.7 µg/g, respectively. After 24 h of exposure, the concentration of Cry2Aa in the diets decreased by 7.42%, and no statistical differences were detected between the values before and after exposure (Student's *t*-test, *P* = 0.185). Therefore, it could be concluded that the Cry2Aa protein in the artificial diets was stable.

The LC_50_ of this batch of Cry2Aa for *P. interpunctella* larvae was 14.9 µg/g fresh weight. Before and after exposure to *C. lividipennis* for 24 h, the artificial diets containing 300 µg/ml Cry2Aa resulted in 90% and 80% mortality of *C. medinalis* larvae, respectively; these mortalities were significantly higher than those that occurred in larvae fed with the pure artificial diet (13%). These results indicated that the Cry2Aa protein in the artificial diets for *C. lividipennis* was bioactive.

**Figure 1:**
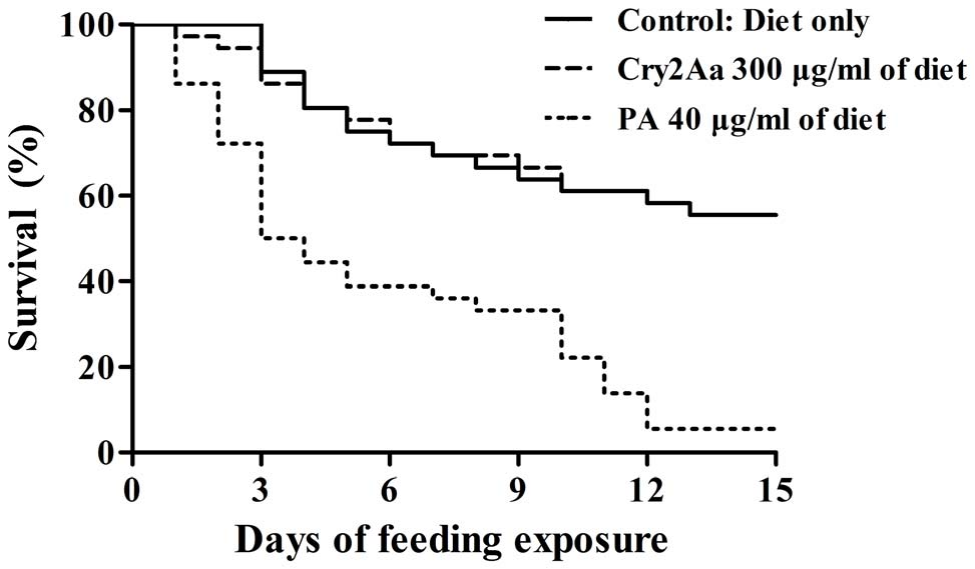
Survival of *Cyrtorhinus lividipennis* fed pure artificial diet or diet containing different insecticidal compounds. 300 µg Cry2Aa and 40 µg PA per ml were incorporated into artificial diets. Pure diet served as a negative control (N = 36).

Survival of the *C. lividipennis* fed with the artificial diet containing 40 µg/ml PA was significantly decreased compared to that of the *C. lividipennis* fed with the pure artificial diet (*P*<0.001) (Fig. 1). This result indicated that the test system employed in the present study could detect the dietary effects of insecticidal compounds. High-dosage exposure to the Cry2Aa protein had no adverse effects on the survival response of *C. lividipennis* in comparison to the pure artificial diet (negative control) (Fig. 1). Similarly, the developmental time from the second instar to adulthood, preimaginal survival and body weight of *C. lividipennis* were unaffected by the Cry2Aa protein in comparison to the negative control (Table 4).

**Table 4:** Effects of purified Cry2Aa incorporating into artificial diet on life-table parameters of *Cyrtorhinus lividipennis*.

Parameters	Treatments		
	Control	300 µg/ml Cry2Aa	40 µg/ml PA
2nd instar developmental time (days ± SE)^a^	2.1±0.15 (35)	2.3±0.13 (35)	2.4±0.21 (23)
3rd instar developmental time (days ± SE)^a^	2.5±0.10 (28)	2.6±0.13(29)	4.2±0.36 (13)**
4th instar developmental time (days ± SE)^a^	3.1±0.17 (21)	3.1±0.24 (22)	7.0±0.58 (3)**
5th instar developmental time (days ± SE)^a^	4.2±0.18 (20)	4.1±0.20 (20)	—
2nd instar-adult developmental time (days ± SE)^a^	11.8±0.40 (20)	11.7±0.40 (20)	—
Preimaginal survival (%)^b^	55.6	55.6	0.0
Male weight (mg ± SE)^c^	0.41±0.02	0.40±0.02	—
Female weight (mg ± SE)^c^	0.58±0.02	0.54±0.02	—

Nymphs of *C. lividipennis* were fed with an artificial diet containing 300 µg/ml Cry2Aa or 40 µg/ml PA (positive control). Pure diet served as a negative control (N = 36). The experiment lasted until adult eclosed. Statistical comparisons were made separately for each of the insecticidal compounds comparing with the control. Asterisks denote significant differences: *P*<0.01.

a Mann–Whitney *U*-test with Bonferroni correction (adjusted α = 0.025).

b Chi-square test with Bonferroni correction (adjusted α = 0.025).

c Student's *t*-test.

### Effects of Cry2Aa on the functional response of *C. lividipennis*

**Figure 2:**
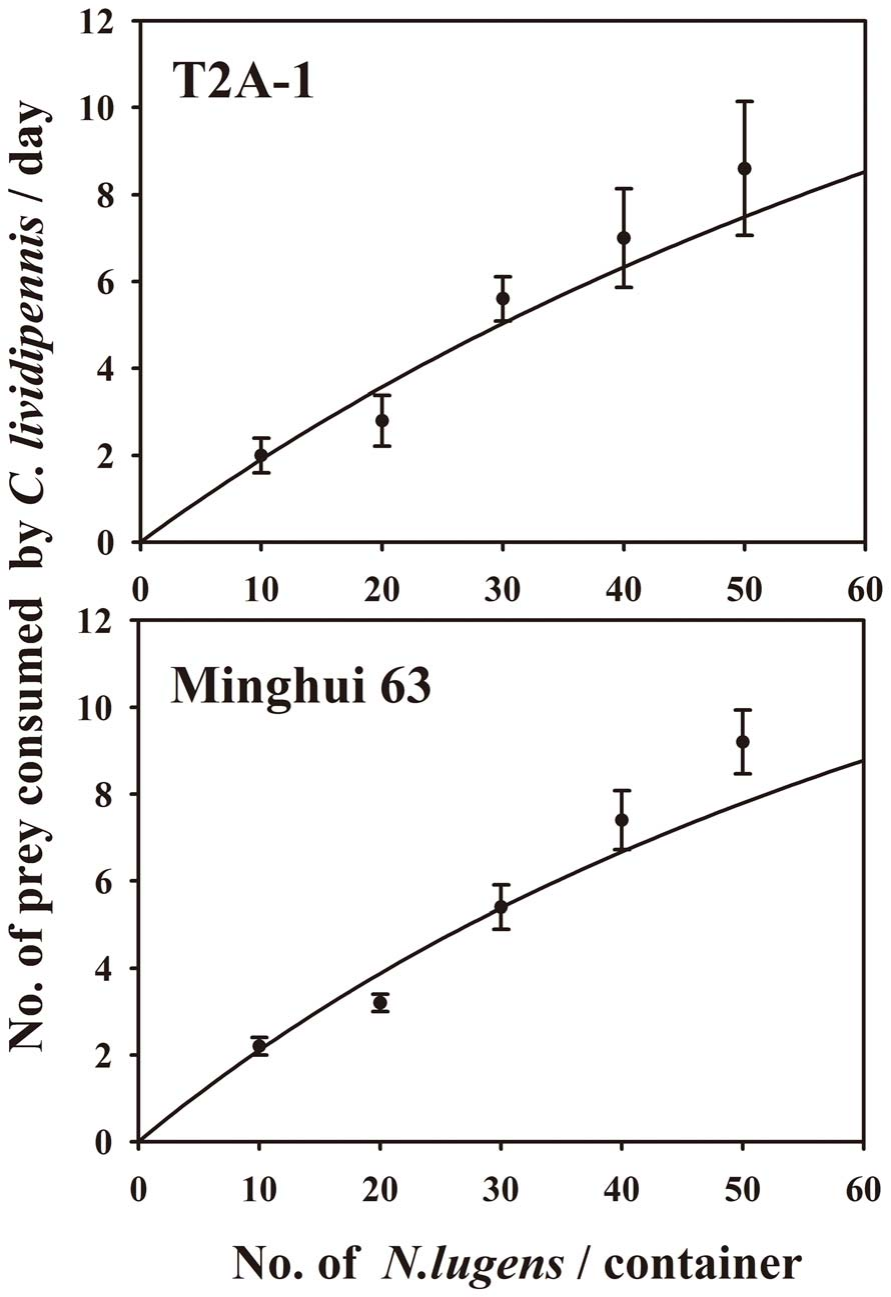
Functional response of *Cyrtorhinus lividipennis* collected from T2A-1 and Minghui 63.

The results indicated that the functional response of *C. lividipennis* to *N. lugens* on both rice lines was typically Type II as described by Holling [32], [33] (Fig. 2). The instantaneous search rate and handling time were not significantly affected by rice line (*P*>0.05) (Table 5).

**Table 5:** Parameters of Type II functional response of *Cyrtorhinus lividipennis* to *Nilapavarta lugens* nymph fed on *Bt* or non-*Bt* rice.

Rice materials	*a*	*Th*	*R^2^* (%)
T2A-1	0.251±0.028	0.036±0.008	95.83
Minghui 63	0.231±0.025	0.037±0.007	95.20

*a*: instantaneous search rate (day^−1^). *Th*: time required to handle a prey (day). Data are represented as mean ± SE.

There was no significant difference between T2A-1 and Minghui 63, based on Student's *t*-test (*P*<0.05).

### Effects of transgenic *cry2Aa* rice on the population density and dynamics of *C. lividipennis*

**Figure 3:**
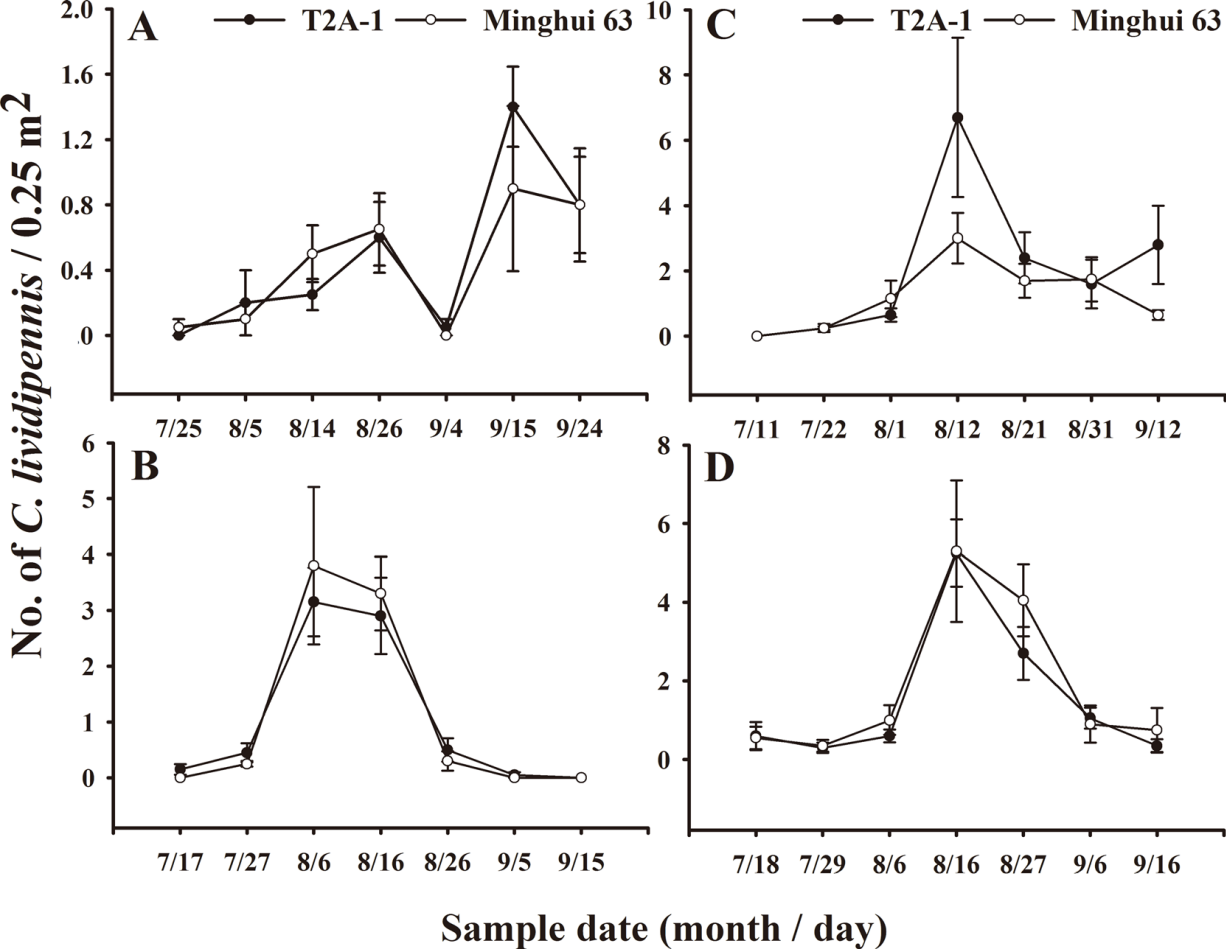
Population dynamics of *Cyrtorhinus lividipennis* collected by vacuum-suction. Data are represented as mean ± SE. (A). Xiaogan, 2011; (B). Xiaogan, 2012; (C). Xiaogan, 2013; (D) Suizhou, 2012. There was no significant difference between *Bt* rice and control plots at the same sampling time, based on Student's *t*-test (N = 4). Repeated measures ANOVA: (A). F_1,6_ = 0.059, *P* = 0.816; (B). F_1,6_ = 0.058, *P* = 0.777; (C). F_1,6_ = 1.366, *P* = 0.287; (D). F_1,6_ = 0.964, *P* = 0.364.

The population density of *C. lividipennis* is shown in Table 6. Compared with that on the nontransgenic Minghui 63, the population density of *C. lividipennis* was not significantly influenced by transgenic *cry2Aa* rice at any site or in any year (Student's *t*-test, *P*>0.05) (Table 6). The population dynamics (means of each sampling date) of *C. lividipennis* are shown in Fig. 3. No significant differences in the population dynamics of *C. lividipennis* were observed between Minghui 63 and transgenic *cry2Aa* rice fields at any sampling date, at any site or in any year (Student's *t*-test, all *P*>0.05). Repeated measures ANOVA analysis showed that the population dynamics were unaffected by rice line (*P*>0.05).

**Table 6:** Population densities (no. of per 0.25 m^2^) of *Cyrtorhinus lividipennis* collected by vacuum-suction.

Rice materials	Xiaogan			Suizhou
	2011	2012	2013	2012
T2A-1	0.47±0.10	1.03±0.09	2.06±0.68	1.55±0.06
Minghui 63	0.43±0.14	1.09±0.20	1.21±0.23	1.84±0.29

N = 4 at both sites in 2011, 2012, and 2013. Data are represented as mean ± SE. There was no significant difference between T2A-1 and Minghui 63 field, based on Student's *t*-test.

## Discussion

The ecological risk assessment of an insect-resistant transgenic crop for a nontarget arthropod (NTA) should be conducted within a tiered scheme: (i) effects on the NTA at elevated doses in a replicated controlled system; (ii) effects on the NTA at realistic doses in a replicated controlled system; (iii) effects on the population of the NTA at realistic doses in a realistic agricultural system [34]. According to these criteria, three experiments were conducted to evaluate the ecological risk of transgenic *cry2Aa* rice to *C. lividipennis*, a primary predator of *N. lugens* that is the main NTA of transgenic *Bt* rice in the present study: (1) a direct feeding experiment, in which *C. lividipennis* was fed an artificial diet containing Cry2Aa at the dose of 10-time higher than that it may encounter in the realistic field condition; (2) a tritrophic experiment, in which the Cry2Aa protein was delivered to *C. lividipennis* indirectly through prey eggs or nymphs; (3) a realistic field experiment, in which the population dynamics of *C. lividipennis* were investigated by vacuum-suction. Direct exposure to elevated doses of the Cry2Aa protein and prey-mediated exposure to realistic doses of the Cry2Aa protein did not result in significant detrimental effects on the developmental time, preimaginal survival, female ratio and body weight of *C. lividipennis*. No significant differences in population density and population dynamics were observed between *C. lividipennis* populations in transgenic *cry2Aa* and nontransgenic rice fields. It could be concluded that transgenic *cry2Aa* rice had no adverse effects on *C. lividipennis*. This study represents the first report of an assessment continuum for the effects of transgenic *cry2Aa* rice on *C. lividipennis*.

Planthoppers are the main nontarget herbivores in transgenic *Bt* rice fields. Determining whether the Bt protein may be transmitted to predators via planthoppers is important for the ecological risk assessment of transgenic *Bt* rice. Several reports have examined Bt protein transmission to predators via predation on planthoppers infesting transgenic *Bt* rice, but their results were inconclusive. Cry1Ab could be transferred to *P. subpiraticus* by predation on *N. lugens* fed on transgenic *cry1Ab* rice, and the content of Cry1Ab in *P. subpiraticus* was significantly higher than that in *N. lugens* fed on transgenic *cry1Ab* rice [19]. Cry1Ab could also be transferred to *U. insecticeps* via predation on *N. lugens* fed on transgenic *cry1Ab* rice, and the concentration of Cry1Ab in *U. insecticeps* was significantly lower than that in *N. lugens* [21]. However, no Cry2Aa was detected in a study of the larvae of a general predator (*C. sinica*) of planthoppers, in which *C. sinica* was provided with *Laodelphax striatellus* fed on transgenic *cry2Aa* rice [24].

In the present study, when *N. lugens* nymphs and T2A-1 seedlings were provided simultaneously to *C. lividipennis*, Cry2Aa was detected in the predator. However, when the *N. lugens* nymphs fed on T2A-1 were removed from the rice and provided alone to *C. lividipennis*, no Cry2Aa was detected in the predator. Similarly, when *N. lugens* eggs and T2A-1 seedlings were provided to *C. lividipennis* simultaneously, Cry2Aa was detected in the predator. However, when the *N. lugens* eggs were removed from the T2A-1 seedlings and provided alone to *C. lividipennis*, no Cry2Aa was detected in the predator. Therefore, it may be inferred that Cry2Aa was not transmitted to *C. lividipennis* via predation on the eggs and nymphs of *N. lugens*; the protein may instead be transferred by the piercing-sucking foraging behavior of *C. lividipennis* on rice. This hypothesis was verified through the results of our supplementary experiments: when *C. lividipennis* nymphs were provided with T2A-1 seedlings alone for 1 day, Cry2Aa was detected in the predator, and the concentration of Cry2Aa in *C. lividipennis* was 17.1±8.6 ng/g (Table 3). Therefore, *C. lividipennis* may serve as a good indicator species in ecological risk assessments of transgenic *Bt* tice.

In the ecological risk assessment of an arthropod-resistant genetically engineered crop, researchers normally use “Tier-1 assays” as the initial step to determine the toxicity of the insecticidal compounds expressed by the transgenic crop on NTAs. In Tier-1 tests, insecticidal compounds are added to artificial diets for the tested NTAs, and the tested organisms are directly exposed to doses of the insecticidal compounds several times higher than those realistically present in the field. Tier-1 tests increase the likelihood that a hazard will be detected if the hazard exists, and therefore provide confidence that minimal risk is present if no adverse effect is detected [34], [35]. Three important factors must be considered in Tier-1 assays: (i) the methods for the delivery of the insecticidal proteins to the test organisms; (ii) the need for and selection of the compounds used as positive controls; and (iii) the methods for monitoring the concentration, stability and bioactivity of the insecticidal proteins during the assay [35]. In the present study, a dietary exposure experiment was conducted in which purified Cry2Aa protein was directly fed to *C. lividipennis* nymphs through its incorporation into a previously described artificial diet for *N. lugens* [29]. This artificial diet was sufficient for sustaining the growth and development of *C. lividipennis* from the second instar to adulthood. Before and after exposure to *C. lividipennis* for 24 h, the Cry2Aa protein in the artificial diets was stable and bioactive. The oral poison PA was used as a positive control to validate our dietary exposure assay. The survival of the *C. lividipennis* fed with the artificial diet containing 40 µg/ml PA significantly decreased compared to that of the *C. lividipennis* fed with the pure artificial diet (*P*<0.001) (Fig. 1). This result confirmed that the test system employed in the current study was able to detect the dietary effects of insecticidal compounds. No detrimental effects on the life-table parameters of *C. lividipennis* were observed when the insects were provided with an artificial diet containing Cry2Aa at a concentration that was nearly ten times higher than that measured in the rice sheaths. This study represents the first report of the use of a Tier-1 system to evaluate the potential effects of Cry2Aa on *C. lividipennis*.

The survival rate of *C. lividipennis* preying the nymphs of *N. lugens* was 32–35%, which was much lower than that of *C. lividipennis* preying eggs of *N. lugens* (87–91%) in the tritrophic experiment. So did body weight of adults of *C. lividipennis*. Similar results have been reported by Chua and Mikil and Chen et al., and they concluded that *N. lugens* eggs was an essential food type for *C. lividipennis*, and *N. lugens* nymphs was not an ideal food for the predator [36], [37]. The different nutrient composition of prey may cause biological parameters difference of predator. *Harmonia axyridis* (Pallas) (Coleoptera: Coccinellidae) is a polyphagous species, the complete development of this predator can be accomplished using the aphid *Acyrthosiphon pisum* (Harris) (Homoptera: Aphididae) or *Ephestia kuehniella* (Zeller) (Lepidoptera: Pyralidae) eggs as substitution prey. Biochemical analyses indicated that amino acids and lipids of *E. kuehniella* eggs were richer than *A. pisum* adults, but, on the contrary, the glycogen of aphids was richer than *E. kuehniella* eggs. Some biological parameters such as larval mortality, adult weight, and fecundity, were modified according to the food eaten [38]. Whether the different survival of *C. lividipennis* in the present study is caused by different nutrient composition of prey needs to be further explored.

## Conclusions

In summary, this comprehensive study, involving a Tier-1 examination system, a tritrophic bioassay, functional response experiments in the laboratory and population dynamics determinations in the field, provides the most complete information to date on the impacts of *Bt* rice expressing Cry2Aa on *C. lividipennis*, a major predator in rice ecosystems. These results indicate that *C. lividipennis* is not sensitive to the Cry2Aa protein and that *Bt* rice (T2A-1) poses a negligible risk to this nontarget organism.
